# Differential solubility of curcuminoids in serum and albumin solutions: implications for analytical and therapeutic applications

**DOI:** 10.1186/1472-6750-8-84

**Published:** 2008-11-06

**Authors:** Wolfgang W Quitschke

**Affiliations:** 1 Department of Psychiatry and Behavioral Science, State University of New York at Stony Brook, Stony Brook, NY 11794-8101, USA

## Abstract

**Background:**

Commercially available curcumin preparations contain a mixture of related polyphenols, collectively referred to as curcuminoids. These encompass the primary component curcumin along with its co-purified derivatives demethoxycurcumin and bisdemethoxycurcumin. Curcuminoids have numerous biological activities, including inhibition of cancer related cell proliferation and reduction of amyloid plaque formation associated with Alzheimer disease. Unfortunately, the solubility of curcuminoids in aqueous solutions is exceedingly low. This restricts their systemic availability in orally administered formulations and limits their therapeutic potential.

**Results:**

Methods are described that achieve high concentrations of soluble curcuminoids in serum. Solid curcuminoids were either mixed directly with serum, or they were predissolved in dimethyl sulfoxide and added as aliquots to serum. Both methods resulted in high levels of curcuminoid-solubility in mammalian sera from different species. However, adding aliquots of dimethyl sulfoxide-dissolved curcuminoids to serum proved to be more efficient, producing soluble curcuminoid concentrations of at least 3 mM in human serum. The methods also resulted in the differential solubility of individual curcuminoids in serum. The addition of dimethyl sulfoxide-dissolved curcuminoids to serum preferentially solubilized curcumin, whereas adding solid curcuminoids predominantly solubilized bisdemethoxycurcumin. Either method of solubilization was equally effective in inhibiting dose-dependent HeLa cell proliferation in culture. The maximum concentration of curcuminoids achieved in serum was at least 100-fold higher than that required for inhibiting cell proliferation in culture and 1000-fold higher than the concentration that has been reported to prevent amyloid plaque formation associated with Alzheimer disease. Curcuminoids were also highly soluble in solutions of purified albumin, a major component of serum.

**Conclusion:**

These results suggest the possibility of alternative therapeutic approaches by injection or infusion of relatively small amounts of curcuminoid-enriched serum. They also provide tools to reproducibly solubilize curcuminoids for analysis in cell culture applications. The differential solubility of curcuminoids achieved by different methods of solubilization offers convenient alternatives to assess the diverse biological effects contributed by curcumin and its derivatives.

## Background

Turmeric is a powder derived from the root of the herb *Curcuma longa *and it is commonly used as a spice and coloring agent. Curcumin is a yellow pigment in turmeric, where it occurs in amounts of 2–9% [1,2]. Although commonly referred to as 'curcumin', commercially available preparations are actually a mixture of the principal ingredient curcumin along with its copurified derivatives demethoxycurcumin and bisdemethoxycurcumin [3]. Therefore, in this report the term 'curcuminoid' will be used to describe such commercially available preparations.

Curcuminoids possess numerous medicinal properties. These include antiinflammatory, antioxidant, antiviral, antiinfective, antimalarial and wound healing properties [4,5]. In cancer related research curcuminoids have been used in cell culture systems, animal models, and clinical trials [6-13]. Curcuminoids have been implicated in inhibiting tumor promotion in skin, oral, intestinal, and colon cancers [6]. It has also been suggested that they inhibit breast cancer metastasis in mice [14,15]. Inhibition of cell proliferation is accomplished by cell cycle arrest or apoptosis [7,16].

The antioxidative effect of curcuminoids and their ability to prevent Aβ aggregation has also attracted attention in Alzheimer disease research [17,18]. For example, orally administered curcuminoids suppressed oxidative damage, synaptophysin loss, and reduced Aβ deposits in rats subjected to intracerebroventricular infusion of Aβ [19]. Orally administered curcuminoids have been implicated in reducing Aβ plaque burden and the amount of soluble Aβ in Alzheimer transgenic mice [20]. In addition, curcuminoids reduced interleukin-1β expression while the level of amyloid protein precursor (APP) remained unchanged [21]. Studies on SH-SY5Y cells showed that curcuminoids counteracted the retinoic acid induced increased expression of APP [22] and nine curcuminoid substances isolated from turmeric had differential effects of protecting PC12 cells from Aβ insult [23]. Another study reported an *in vitro *dose dependent inhibition of Aβ40 aggregation and disaggregation of Aβ40 fibrils with curcuminoid IC_50 _values of 0.8 μM and 1 μM, respectively. Similar results were obtained for curcuminoid-mediated inhibition of Aβ fibril formation and extension, as well as fibril destabilization with EC_50 _values ranging from 0.19 to 0.63 μM [24]. The toxicity of Aβ to differentiated SH-SY5Y cells was inhibited at a 1 μM curcuminoid concentration. Furthermore, peripherally injected curcuminoids were found to cross the blood-brain barrier and orally administered curcuminoids to Tg2576 mice reduced amyloid burden and plaque formation [25].

Despite such encouraging reports, the study of curcuminoids is severely limited by their exceedingly low bioavailability following oral administration. This is largely a consequence of their extremely poor solubility and instability in aqueous solutions, in particular at alkaline pH [26,27]. However, curcuminoid stability was increased in human blood and in tissue culture media containing 10% fetal calf serum (FCS) [28]. In clinical trials with oral administration of curcuminoids, the observed circulating blood or plasma concentrations were found to range from either undetectable to about 1 μM [29-32]. Similar results were observed in the rat [33]. In contrast, curcuminoids are soluble to variable degrees in a number of organic solvents. Curcuminoids are often prepared as a stock solution in one of these solvents, typically dimethyl sulfoxide (DMSO) or methanol. After administering DMSO-dissolved curcuminoids to mice by intraperitoneal injection, initial plasma concentrations were as high as 6 μM [34]. In cell culture studies, the curcuminoid stock solution is usually added to the cell culture medium at various final concentrations. Unfortunately, upon adding such predissolved curcuminoids to an aqueous solution they precipitate. Therefore, the final concentration of the actual soluble curcuminoids in the medium remains inferential, since it is based on the dilution of the total amount added. Methods are here described that differentially solubilize high concentrations of biologically active curcuminoids in serum. This provides a novel method for curcuminoid administration in therapeutic applications and it allows for the preparation of tissue culture media with defined concentrations of differentially solubilized curcuminoids.

## Methods

### Reagents and solutions

BSA [Fraction V, 96–99% albumin], curcuminoids ('curcumin' > 95%) [Fluka], and DMSO [ACS reagent] were obtained from Sigma Chemical Company. 1-butanol (HPLC grade) was purchased from Fisher Scientific. Fetal calf, horse, rat, human and rabbit sera were supplied by Aleken Biologicals. DMEM (high glucose), 0.05% Trypsin-EDTA, and Penicillin-Streptomycin (10,000 units/ml, 100×) were provided by GIBCO (Invitrogen). Acetonitrile and water (both HPLC grade) were obtained from Mallinckrodt Baker Inc. All other standard laboratory chemicals were purchased from Research Organics.

BSA was prepared as 10% or 20% (w/v) stock solutions in PBS (137 mM NaCl, 10 mM Phosphate, 2.7 mM KCl, pH 7.4) and used directly or diluted as indicated. Sera were thoroughly mixed and used as provided. Butanol was equilibrated with an excess of deionized water and used after phase separation. Eluent solutions A (5% acetonitrile, 0.01% ammonium acetate, pH 4.5, and 95% water) and B (95% acetonitrile, 0.01% ammonium acetate, pH 4.5, and 5% water) were used to generate a gradient in reversed phase chromatography (see below).

### Curcuminoid solubilization and cell culture media preparation

Either solid curcuminoid powder in varying amounts (1–70 mg) or 10 μl of curcuminoids (10–500 mM) predissolved in DMSO were added to 1 ml of serum or BSA solutions in 1.5 ml microfuge tubes. Solid curcumin was vortexed until the powder was well suspended. When DMSO-dissolved curcuminoids were added to the aqueous protein solution, much of the curcuminoids precipitated and the precipitate was vortexed until suspended. Time course experiments showed that no additional curcuminoid solubilization occurred after 4–6 h of incubation of the suspensions at 4°C. However, to ensure that complete equilibrium was established, incubation was continued on a rotary mixer for 16–24 h. Thereafter, the remaining insoluble curcuminoids were pelleted by centrifugation for 10 min at 14,000 × g. Aliquots of 200 μl were withdrawn for butanol extraction. In all cases, extreme care had to be exercised not to include insoluble curcuminoids in samples designated for butanol extractions, since this would substantially increase the apparent amount of soluble curcuminoids.

For cell culture media preparation, 29 ml of FCS were either mixed with 870 mg (30 mg/ml) of solid curcuminoids or 145 μl (5 μl/ml) of DMSO containing 500 mM curcuminoids. In both cases, the suspended curcuminoids were stirred in 50 ml Erlenmeyer flasks at 4°C for 16–24 h. Residual insoluble curcuminoids were removed by two centrifugations at 14,000 × g. From the resulting FCS-curcuminoid solution, 1 ml was collected for butanol extraction and spectrophotometric concentration determination, or reversed phase chromatography. Of the remaining FCS-curcuminoid solution, 25 ml were added to 500 ml of DMEM. The resulting media were sterilized through 0.45 μm filters (Pall Life Sciences). Aliquots of 0.5 ml were then removed for butanol extraction and concentration determination. Curcuminoid-containing media were diluted with standard medium to obtain the desired final curcuminoid concentrations.

In experiments addressing the influence of mixing technique on curcuminoid solubility, solid curcuminoids were added to 10 ml of FCS (30 mg/ml) and mixed either by magnetic stirring at medium speed in a 25 ml Erlenmeyer flask, or by rotation on a rotational mixer for 16–24 h. Alternatively, DMSO-dissolved curcuminoids (500 mM) were added to 10 ml of FCS (5 μl/ml) and similarly mixed.

### Spectrophotometric concentration determinations and curcuminoid extractions

**Figure 1:**
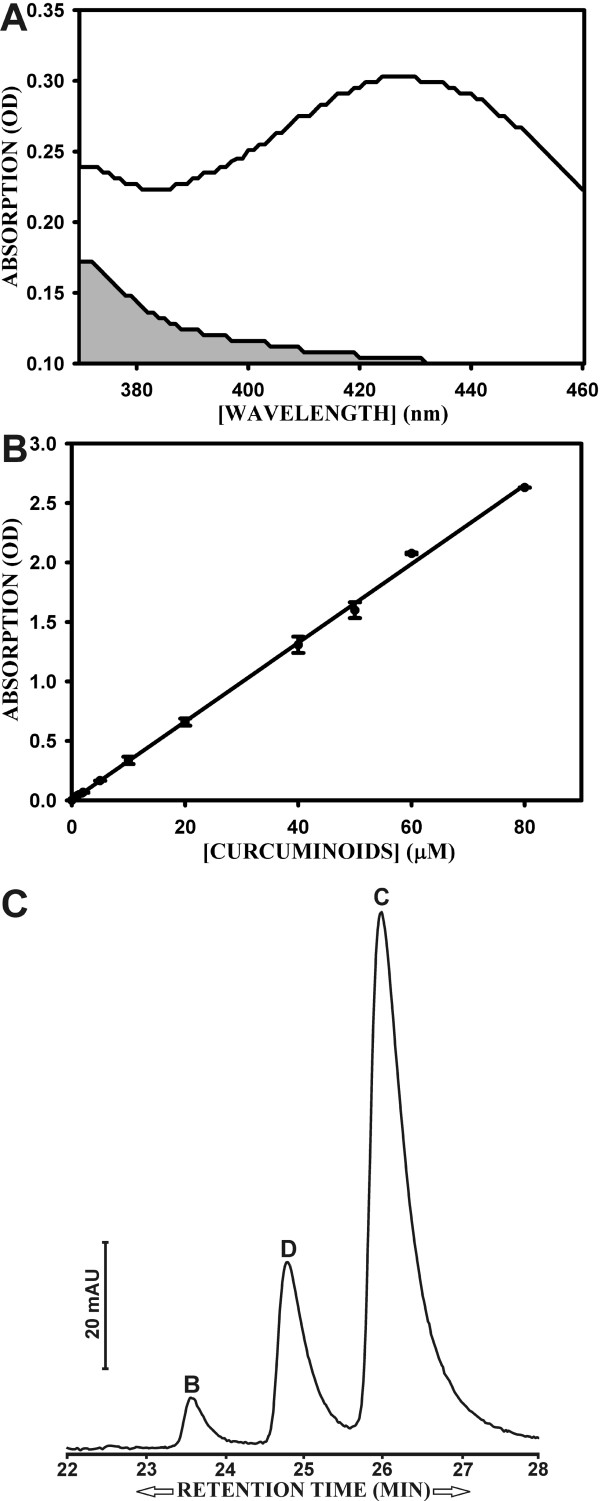
**Spectral absorption of curcuminoids solubilized in butanol**. A) Spectral scan of 6 uM curcuminoids in water-saturated 1-butanol (white area) between wavelength 370 and 460 nm with increments of 1 nm. The gray area delineates the background absorption of water-saturated butanol. B) Dose curve of curcuminoids (1–80 μM) solubilized in water-saturated butanol. Data are presented as absorption (OD) as a function of curcuminoid concentration (μM) at a wavelength of 427 nm. C) Elution profile after reversed phase chromatography of 50 μM curcuminoids dissolved in 27.5% acetonitrile solution (see Methods). The vertical bar represents 20 milliabsorption units (mAU). Elution peaks of curcuminoids at indicated retention times (rt) designate bisdemethoxycurcumin (B), demethoxycurcumin (D), and curcumin (C).

A spectral scan was generated with a 6 μM solution of curcuminoids in water-saturated butanol, which was obtained by diluting a 500 mM stock solution of curcuminoids dissolved in DMSO. The background absorption scan was derived from water-saturated butanol without curcuminoids. Aliquots of 200 μl were placed in a 96-well microplate and scanned at 1 nm increments between wavelengths of 370 and 460 nm in a μ Quant microplate spectrophotometer (Bio-Tek Instruments) [Fig. 1A]. All subsequent curcuminoid concentration determinations were carried out at an absorption wavelength of 427 nm and the background subtracted from the resulting optical density (OD). A dose curve was established to correlate spectral absorption with curcumin concentration.

Curcuminoids were extracted from 200 μl serum or BSA solutions by mixing with 1 ml of water-saturated butanol. Water-saturated butanol was used instead of pure butanol because water partitions into the butanol phase at a volume ratio of slightly more then 1/10. The biphasic solution was thoroughly vortexed and phases were separated by microcentrifugation. Aliquots of 200 μl of the upper butanol phase were removed and either added directly to a 96-well microplate (Corning Inc.) or further diluted with butanol at ratios of 1:5 or 1:10 to achieve OD values within the linear absorption range. The background OD readings were uniformly about 0.1 (+/- 0.01) OD units and these values were subtracted from those obtained with the extracted curcuminoid solutions. Because of the lower concentration of curcuminoids in cell culture media, aliquots of 0.5 ml were extracted with 1 ml of butanol. In both cases, more than 95% of the curcuminoids uniformly partitioned into the organic phase. However, DMEM and other tissue culture media typically contain the indicator dye Phenol Red, which also partially partitions into the butanol phase. This resulted in higher background readings of about 0.220 OD (+/- 0.02) units. These higher background readings that in these instances were subtracted from the experimental values did not appreciably affect measurement accuracy or precision.

### Cell culture and curcuminoid stability in media

HeLa cells (ATCC#: CCL-2) were grown in DMEM containing 5% FCS and penicillin-streptomycin (100 units/ml) at 37°C in an incubator equilibrated with 5% CO_2_. For cell survival analysis, cells were seeded at about 20% confluence in 25 cm^2 ^flasks (Sarstedt). Cells were photographed in three random viewing fields using phase contrast microscopy (Minolta) and counted. The number obtained from the initial cell count was designated as 100%. Thereafter, cells were incubated in media containing 0, 10, 20, 30, 40, and 50 μM soluble curcumin. Media were changed and cells counted daily during three days of incubation.

The stability of soluble curcuminoids in cell culture media stored at 4°C was measured by extracting 0.5 ml aliquots of media with 1 ml of butanol at indicated time intervals between 0 and 28 days after preparation. Alternatively, 10 ml of media were stored in 25 cm^2 ^flasks at 37°C in a tissue culture incubator, either with or without CO_2 _exposure. Aliquots of 0.5 ml were removed for curcuminoid extractions at intervals ranging from 0 to 9 days. Background measurements were subtracted from identically incubated media devoid of curcuminoids.

### Sequential addition of solid and DMSO-dissolved curcuminoids to 5% BSA and FCS

Either 36 mg of solid curcuminoids or 12 μl of 500 mM DMSO-dissolved curcuminoids were added to 1.2 ml of 5% BSA or FCS and processed as described. Thereafter, the insoluble curcuminoids were pelleted by centrifugation and the entire supernatant removed. Aliquots of 200 μl were butanol extracted for total curcuminoid determination and 20 μl were extracted for reversed phase chromatography. The remaining supernatants were mixed with either 30 mg of solid curcuminoids or 10 μl of 500 mM DMSO-dissolved curcuminoids in reverse order. The suspensions were reincubated and then processed for concentration determination.

### Sequential extraction of curcuminoids from 5% BSA

Solid curcuminoids in amounts of 1, 10, 20, 30, 40, and 50 mg were added to 1 ml of 5% BSA and incubated as described above. Thereafter, the insoluble curcuminoids were pelleted by centrifugation and the entire supernatant removed, followed by adding 1 ml of fresh 5% BSA to the remaining pellet. Aliquots of 200 μl were extracted with 1 ml of butanol and the concentration of total soluble curcuminoids was determined. Where applicable, 20 μl aliquots were analyzed by reversed phase chromatography. This process was repeated 10 times. After the last extraction, the remaining insoluble curcuminoid pellets were washed three times with deionized water and dried under vacuum. The dried pellets were then dissolved in 200 μl DMSO. Aliquots were diluted in butanol and the yield of curcuminoids in the final pellet determined spectrophotometrically. Aliquots of 10 μl from the respective DMSO-dissolved final pellets were then added to 1 ml of 5% BSA and solubilized as described above for concentration determination and reversed phase chromatography.

### Reversed phase chromatography of soluble curcuminoids

Curcuminoids solubilized in protein solutions (20 μl) or media samples (200 μl) were extracted for reversed phase chromatography with 300 μl of water-saturated butanol and evaporated in a Savant Speed-Vac concentrator attached to a vacuum pump. The dried residue was reconstituted in a 0.5 ml solution containing 75% eluent A (5% acetonitrile, 0.01% ammonium acetate, pH 4.5, and 95% water) and 25% eluent B (95% acetonitrile, 0.01% ammonium acetate, pH 4.5, and 5% water), representing an acetonitrile concentration of 27.5%. Reversed phase chromatography was performed on an FPLC Äkta Purifier system equipped with a Source 5RPC ST 4.6/150 column (GE Healthcare). Reconstituted curcuminoids (300–400 μl) were loaded onto a 100 μl sample loading loop and separated with a 35 ml linear gradient, which ranged from a starting concentration of 75% eluent A and 25% eluent B to a final concentration of 15% eluent A and 85% eluent B (27.5% – 81.5% acetonitrile) at a flow rate of 1 ml/min. The eluent was monitored at a wavelength of 427 nm. Quantitation of individual peaks was carried out with the Unicorn (version 2.2) peak integration program.

### Concentration calculations and statistical analysis

The arithmetic mean of the molecular masses of the three curcuminoids (338 kDa) was used as a general reference value for spectrophotometric concentration calculations. This was necessary since the relative contribution of each curcuminoid varied considerably between different experimental conditions. In addition, the molar absorptivity (ε) of the three curcuminoids dissolved in ethanol were shown to vary from 6.73 (x10^4 ^L cm^-1^mol^-1^) for curcumin to 4.95 for bisdemethoxycurcumin at 425 nm [35]. The molar absorptivity for the curcuminoid preparation solubilized in butanol was essentially the same as that dissolved in ethanol (data not shown). Hence, it is likely that similar variabilities in molar absorptivity between the three curcuminoids dissolved in butanol exist. However, using the arithmetic mean as a molecular mass reference assured that the reported concentration values were within 15% of the actual concentrations, regardless of the relative distribution of the three curcuminoids within the measured sample.

All data points were calculated as the average of at least three independent experiments, each assayed in duplicate. Error bars represent the standard deviation from the average. Where applicable, the concentration of soluble curcumin as a function of added curcumin was fitted to an exponential saturation function with Sigma Plot software.

## Results and discussion

### Spectral properties of curcuminoids solubilized in butanol

Spectrophotometry was used to systematically determine the concentration of soluble total curcuminoids in serum. Since this required a reliable dose curve, it was essential to solvent-extract the solubilized curcuminoids from the aqueous solutions in a quantitative manner. An ideal solvent proved to be water-saturated butanol, which has the capacity to solubilize curcuminoids up to an approximately 9.5 mM concentration. This solvent is relatively nontoxic and it has a low vapor pressure, which eliminates concerns about volume changes due to evaporation during the assay. It is also chemically inert to the polystyrene used in microplates. After the first extraction of 200 μl FCS-solubilized curcuminoids with 1 ml of butanol, more than 95% of the soluble curcuminoids partitioned into the butanol phase.

A spectral scan between wavelengths 370 nm and 460 nm produced a maximum absorption plateau between wavelengths 425 nm and 431 nm (Fig. 1A). A wavelength of 427 nm was selected for concentration determinations. At this wavelength absorption was linear at curcuminoid concentrations between 2 and 80 μM corresponding to a maximum optical density (OD) of about 2.5 (Fig. 1B).

A 50 μM solution of Curcuminoids in butanol was further analyzed by reversed phase chromatography. Three sequential peaks were eluted that were identified as curcumin (retention time (rt): 26 min), demethoxycurcumin (rt: 24.8 min), and bisdemethoxycurcumin (rt: 23.6 min) [Fig. 1C]. The identification was based on elution profiles obtained with similar acetonitrile gradients [36-38] and on the relative amount of each component present in the curcuminoid mixture. Integrating the areas under the peaks resulted in relative ratios of 79% curcumin, 18% demethoxycurcumin, and 3% bisdemethoxycurcumin. These values are in the same range as those reported for other commercial preparations [29,39].

### The solubility of curcuminoids in FCS differs depending on whether they are added in solid or DMSO-dissolved form

**Figure 2:**
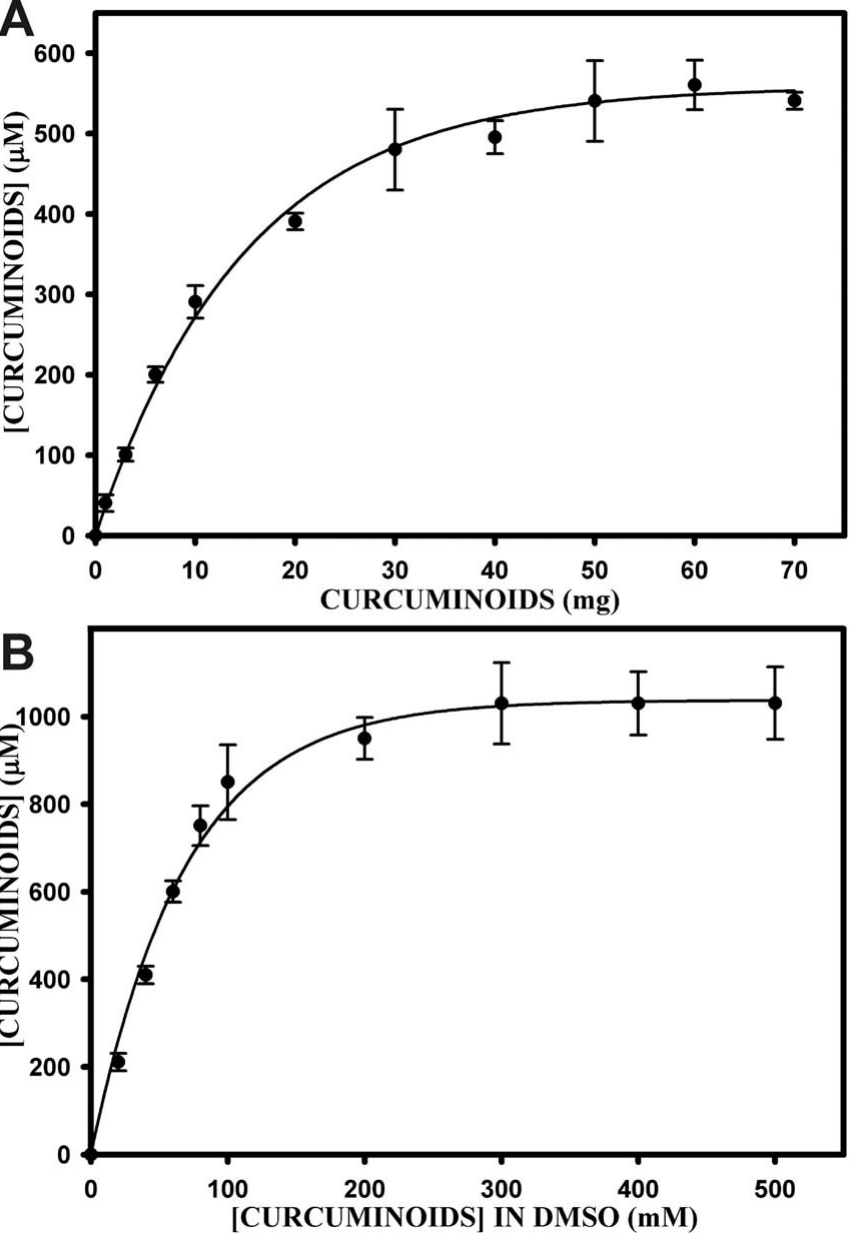
**Solubility of curcuminoids in FCS**. A) Total curcuminoid solubility (μM) in 1 ml of FCS as a function of adding increasing amounts (1–70 mg) of solid curcuminoids. B) Curcuminoid solubility (μM) in 1 ml of FCS as a function of adding 10 μl aliquots of DMSO-dissolved curcuminoids at concentrations ranging from 10 to 500 mM.

Using these spectrophotometric parameters, a systematic analysis of the solubility of curcuminoids in FCS was carried out (Fig. 2). Powdered solid curcuminoids in amounts ranging from 1 mg to 70 mg were added to 1 ml of FCS (Fig. 2A). The concentration of soluble total curcuminoids in FCS increased linearly to about 10 mg of added curcuminoids. When more than 30 mg of solid curcuminoids were added, saturation occurred at concentrations of about 500–600 μM FCS-soluble curcuminoids. Since a concentration of 500 μM represented only about 168 μg/ml of curcuminoids in solution, only a minute fraction (< 1%) of the added solid curcuminoids (30–70 mg) was solubilized in FCS.

To determine whether adding a defined concentration of curcuminoids predissolved in an organic solvent would alter the final solubility in FCS, 10 μl of DMSO-dissolved curcuminoids at concentrations ranging from 20 mM to 500 mM were added to 1 ml of FCS (Fig. 2B). Compared to the results obtained by adding solid curcuminoids, the solubility in FCS almost doubled to over 1000 μM at saturation. Near-saturation levels of about 850 μM FCS-soluble curcuminoids were achieved with 10 μl of 100 mM DMSO-dissolved curcuminoids. This corresponded to about 0.37 mg of total curcuminoids added. This was more than 80-fold less than the comparable 30 mg of solid curcuminoids required for near-maximum FCS-solubility (Fig. 2A). Within the linear range of the saturation curve for DMSO-dissolved curcuminoids (20–100 mM), the amount of FCS-soluble curcuminoids corresponded to about 70–85% of the total added curcuminoids. This value was about 100-fold higher than that obtained by adding solid curcuminoids (Fig. 2A). This indicated that predissolving curcuminoids in DMSO rendered them in a physical form that was more favorable for solubilization in FCS than the solid powder.

### Curcuminoids are soluble in bovine serum albumin (BSA) solutions

**Figure 3:**
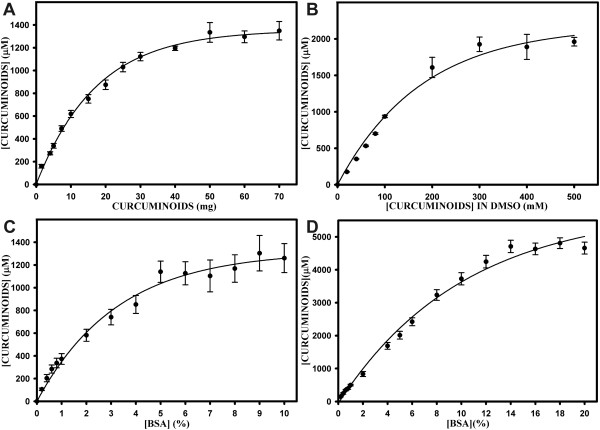
**Solubility of curcuminoids in BSA solutions**. A) Curcuminoid solubility (μM) in 1 ml of 5% BSA as a function of adding increasing amounts (1–70 mg) of solid curcumin. B) Curcuminoid solubility (μM) in 1 ml of 5% BSA as a function of adding 10 μl aliquots of DMSO-dissolved curcuminoids at concentrations ranging from 10 to 500 mM. C) Curcuminoid solubility (μM) as a function of adding 30 mg solid curcuminoids to 1 ml of BSA solutions ranging in concentrations from 0.2 to 10%. D) Curcuminoid solubility (μM) as a function of adding 10 μl aliquots 500 mM DMSO-solubilized curcuminoids to 1 ml of BSA solutions ranging in concentrations from 0.2 to 20%.

Since albumin is a major component of total serum protein, the solubility of curcuminoids in BSA solutions was also examined. BSA was dissolved in phosphate-buffered saline (PBS) at a concentration of 5%. One milliliter of this solution was then incubated with solid curcuminoids in amounts ranging from 1–70 mg (Fig. 3A). The solubility of solid curcuminoids exhibited saturation kinetics similar to those observed with FCS. However, saturation levels of soluble curcuminoids in 5% BSA were attained at concentrations of about 1200–1400 μM. These concentrations were more than twice as high as those achieved with FCS (Fig. 2A) and they represented a molar ratio of curcuminoid/BSA of about 1.6–1.9. A similar saturation curve was observed by adding increasing amounts of DMSO-dissolved curcuminoids to 5% BSA solutions (Fig. 3B). In this case, saturation occurred at curcuminoid concentrations of about 2000 μM, which represented a molar curcuminoid/BSA ratio of about 2.7. Hence, the concentrations at saturation obtained by adding either solid or DMSO-dissolved curcuminoids to 5% BSA were about two-fold higher than those achieved with FCS (Fig. 2).

When the constant amount of 30 mg solid curcuminoids was added to 1 ml of solutions with increasing concentrations of BSA (0.2–10%), saturation was observed at about 5% BSA yielding a soluble curcuminoid concentration of about 1200 μM, which again represented a molar curcuminoid/BSA ratio of 1.6 (Fig. 3C). However, at BSA concentrations of less than 1%, curcuminoid/BSA ratios as high as 3.3 were calculated. At higher BSA concentrations the saturation kinetics indicated that the amount of solid curcuminoids available for solubilization in BSA was the limiting factor, although at a 1400 μM curcuminoid concentration only about 1.7% of the total added curcuminoids had actually been solubilized.

In contrast, when 10 μl of DMSO-dissolved curcuminoids at a constant 500 mM concentration were added to solutions with increasing concentrations of BSA, the BSA-soluble curcuminoid concentration increased to a maximum of about 4500 μM, which was obtained at BSA concentrations above 14% (Fig. 3D). Within the linear range of the curve, the molar curcuminoid/BSA ratio remained constant at about 2.6. These curcuminoid/BSA ratios differ somewhat from those reported by Barik et al. [40]. In that study, the interaction of curcumin and BSA was studied by fluorescence spectroscopy. The authors found that curcumin bound at high affinity to BSA within a microdomain that is largely nonpolar. Their data suggested at least one, but possibly two binding sites of curcumin in BSA. The data reported here suggest between two and four BSA binding sites for curcumin or its derivatives. These discrepancies may be accounted for by differences in the methods of solubilization or the concentration of curcuminoids in solution.

At saturating BSA concentrations (14–20%), the amount of DMSO-dissolved curcuminoids converted into BSA-solubilized curcuminoids was about 92–96% (Fig. 3D), whereas the comparative value attained with solid curcuminoids was about 1.7% (Fig. 3A). This again demonstrated that DMSO-dissolved curcuminoids were far more effective for solubilization than solid curcuminoids in both FCS and BSA solutions.

### Reversed ratios of solid and DMSO-dissolved curcuminoids in FCS and BSA solutions

**Figure 4:**
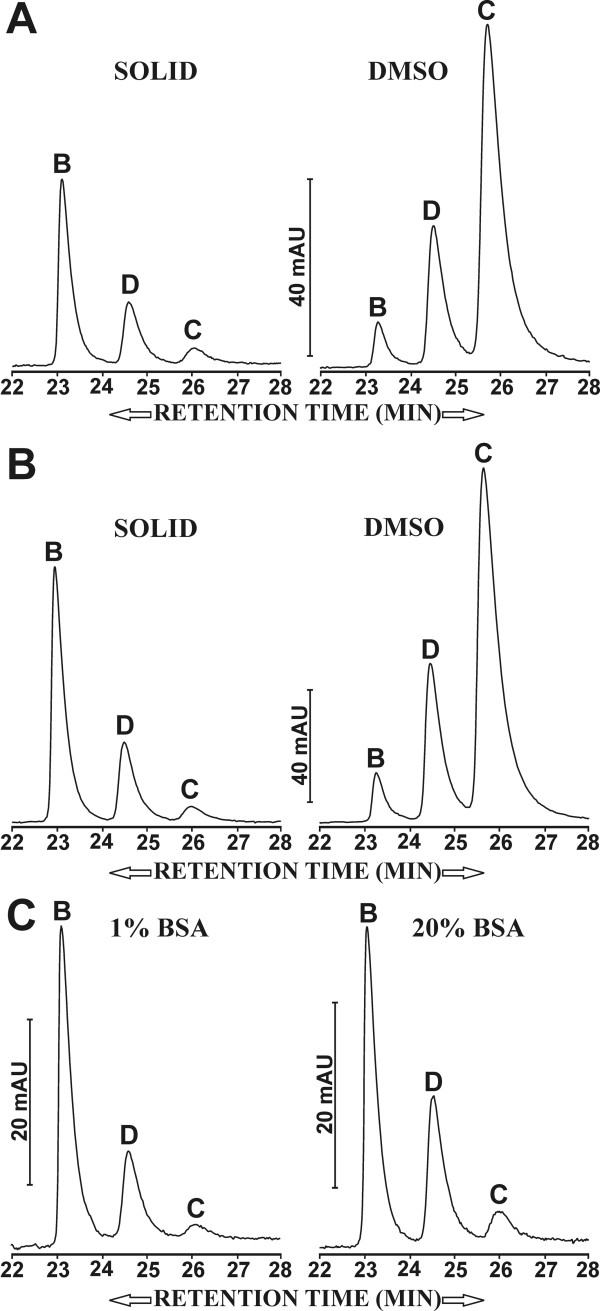
**Elution profiles of curcuminoids separated by reversed phase chromatography**. A) 30 mg of solid (left profile) curcuminoids or 10 μl of 500 mM DMSO-dissolved (right profile) curcuminoids solubilized in 1 ml of FCS. B) 30 mg of solid (left profile) curcuminoids or 10 μl of 500 mM DMSO-dissolved (right profile) curcuminoids solubilized in 1 ml of 5% BSA. C) 30 mg of solid curcuminoids solubilized in 1 ml of 1% BSA (left profile) or 20% BSA (right profile). Vertical bars represent mAUs and individual curcuminoids are designated as described in Fig. 1C.

The differential solubility of curcuminoids in FCS and BSA solutions was also analyzed by reversed phase chromatography (Fig 4). When DMSO-dissolved curcuminoids were solubilized in either FCS or 5% BSA, their relative concentrations declined in the order: curcumin (72%) > demethoxycurcumin (23%) > bisdemethoxycurcumin (5%) [Fig. 4A,B]. These soluble curcuminoid ratios were largely a reflection of the ratios in the DMSO stock solution (Fig. 1C). In contrast, when 5% BSA or FCS was incubated with solid curcuminoids, the elution profile of the solubilized curcuminoids was effectively reversed. Here, the major solubilized curcuminoid was bisdemethoxycurcumin (71%) followed by demethoxycurcumin (26%) and curcumin (3%) [Fig. 4A,B]. The effect of solubilizing 30 mg of solid curcuminoids on elution profiles was also examined when the BSA concentrations were increased from 1% to 20%. However, this increase in BSA concentration produced only modest changes in the curcuminoid ratios. The relative amount of bisdemethoxycurcumin declined from 76% with 1% BSA to 64% with 20% BSA, which was reflected in a concomitant increase in the relative amount of demethoxycurcumin from 23% to 32%, and of curcumin from 1% to 4% (Fig 4C). These results show that the different methods of solubilizing curcuminoids not only produced quantitative differences in the total amounts solubilized, but they also resulted in profound changes in the relative concentrations of the individual curcuminoids.

### The solubility of curcuminoids in 5% BSA and FCS depends on the order of addition of DMSO-dissolved and solid curcuminoids

**Figure 5:**
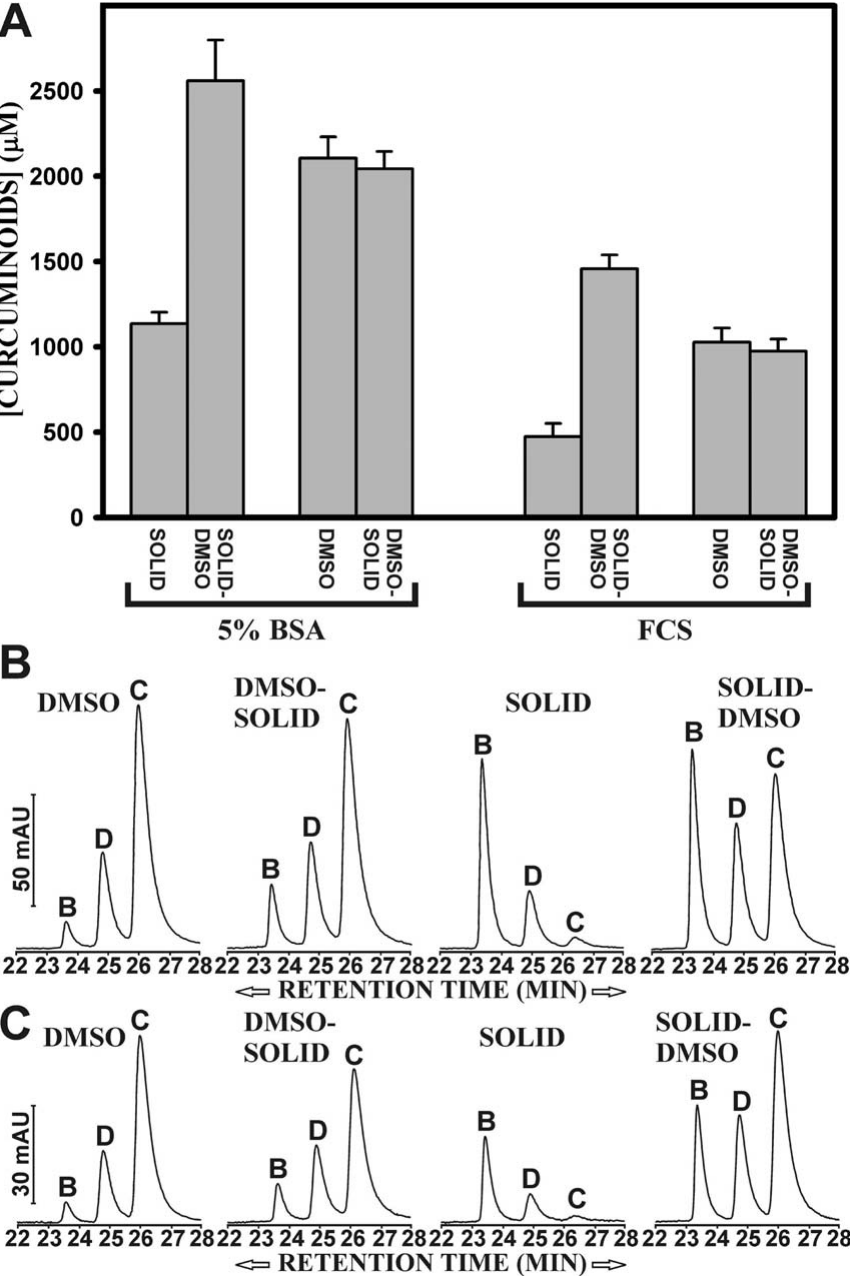
**Sequential addition of curcuminoids to 5% BSA or FCS**. A) Solid or DMSO-dissolved curcuminoids were added individually or sequentially to 5% BSA (left bracket) or FCS (right bracket) as described in Methods. After each addition the concentration of total soluble curcumin in the protein solutions was determined. B) Elution profiles of curcuminoids after single (DMSO, SOLID) or sequential (DMSO-SOLID, SOLID-DMSO) addition of DMSO-dissolved (DMSO) or solid (SOLID) curcuminoids to 5% BSA. C) Elution profiles of curcuminoids after single or sequential addition of DMSO-dissolved or solid curcuminoids to FCS. Vertical bars represent mAUs and individual curcuminoids are designated as described in Fig. 1C.

This differential solubility of the curcuminoids was further investigated by sequentially incubating 5% BSA or FCS with either 30 mg of solid curcuminoids or with 10 μl of 500 mM DMSO-dissolved curcuminoids. When 5% BSA or FCS was first incubated with solid curcuminoids followed by incubation with DMSO-dissolved curcuminoids, a several-fold increase in the concentration of BSA- and FCS-solubilized curcuminoids was achieved (Fig. 5A). This level of curcuminoid solubility was higher than that obtained by adding DMSO-dissolved curcuminoid alone and it implied that the two additions were cumulative. Conversely, when FCS or 5% BSA was first incubated with DMSO-dissolved curcuminoids followed by solid curcuminoids, the final concentration of soluble curcuminoids remained essentially constant.

The sequentially solubilized curcuminoids in FCS and 5% BSA were also analyzed by reversed phase chromatography. The elution profiles showed a similar pattern for both 5% BSA and FCS (Fig. 5B,C). After the initial incubation with DMSO-dissolved curcuminoids followed by incubation with solid curcuminoids, there was a small increase in the relative solubility of bisdemethoxycurcumin and demethoxycurcumin. However, this increase was not nearly as high as would be expected from the sum of the incubations with solid and DMSO-dissolved curcuminoids alone (Fig. 5B,C). Concomitantly, there was a small decrease in the relative amount of solubilized curcumin.

In contrast, when the protein solutions were first incubated with solid curcuminoids followed by DMSO-dissolved curcuminoids the elution profile changed dramatically. The relative levels of bisdemethoxycurcumin and demethoxycurcumin were essentially the cumulative sum of the individually added solid and DMSO dissolved curcuminoids in both FCS and 5% BSA. However, the relative solubility of curcumin following sequential addition was only consistent with the sum of the individual additions to FCS (Fig. 5B). By comparison, the relative contribution of curcumin in 5% BSA was lower after the sequential additions than would be expected from the sum of adding DMSO-dissolved and solid curcumin individually. The reason for this difference might be due to the heterogeneous protein composition of FCS, which may result in multiple proteins contributing to curcuminoid solubility. The data obtained by reversed phase chromatography are in agreement with the spectrophotometrically determined solubility of total curcuminoids (Fig. 5A).

These results affirm that the mechanisms for converting solid and DMSO-dissolved curcuminoids into BSA- and FCS-soluble curcuminoids are profoundly different. The reason for these differences is uncertain, in particular since a major portion of the DMSO-dissolved curcuminoids initially precipitate upon addition to the aqueous protein solution. It is conceivable that this is due to differences in structure between the added solid curcuminoids and the curcuminoids precipitated from the DMSO solution. Commercial curcuminoids are commonly produced by the extraction of turmeric powder with a range of organic solvents followed by evaporation [41]. Such a process is likely to yield ordered curcuminoid crystals or polycrystalline structures. Solid curcuminoids would thus allow only limited surface access of the solvent to disrupt the defined structures. In contrast, a precipitation would be considered a disorganized event yielding an amorphous solid with solvent molecules trapped between the particles. Hence, the combination of increased surface area for solvent access and disorganized structure would favor enhanced solubilization of DMSO-dissolved curcuminoids. It is possible that this phenomenon is related to observations made with solid dispersion experiments using mixtures of polyvinylpyrrolidine (PVP) and curcumin at different ratios [42]. In that study, pure curcumin displayed crystalline structures with well-developed edges. In contrast, solid dispersions with varying amounts of PVP yielded spherical particles with increased surface area. Similarly, the solubility of curcumin in aqueous medium increased dramatically with solid dispersions at higher PVP/curcumin ratios [42].

### Sequential extraction of solid curcuminoids reduces soluble yield and changes curcuminoid ratios

**Figure 6:**
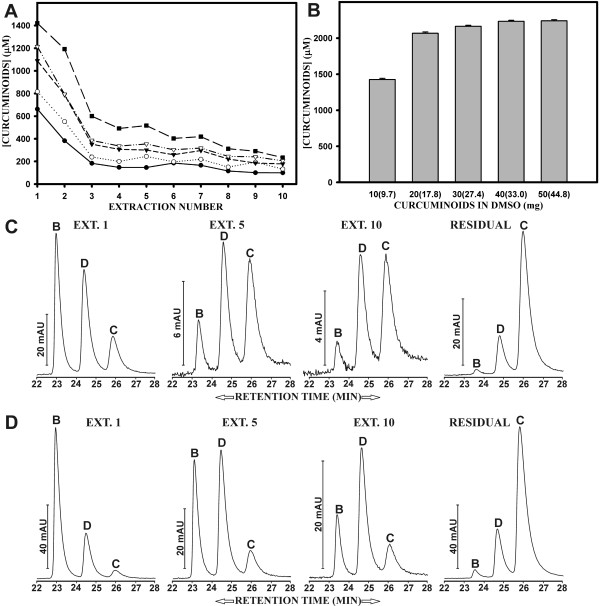
**Sequential extraction of solid curcuminoids with 5% BSA**. A) Solid curcuminoids in amounts of 10 mg (●), 20 mg (○), 30 mg (▼), 40 mg (▽), and 50 mg (■) were added to 1 ml of 5% BSA. After centrifugation, the supernatant containing BSA-soluble curcuminoids was removed and fresh 5% BSA was added to the remaining pellets containing insoluble curcuminoids (Methods). The procedure was repeated ten times and the concentration (μM) of total soluble curcuminoids determined. B) After the tenth extraction of solid curcumin with 5% BSA, the final pellets were washed and dissolved in 200 μl DMSO. A 10 μl aliquot of the DMSO-dissolved curcumin was added to 1 ml of 5% BSA and processed for concentration determination. The bars represent the concentration (μM) of total curcuminoids solubilized in 5% BSA. The numbers under the bars indicate the starting amount (mg) of the curcuminoids and the numbers in parentheses the final yield (mg) of DMSO-dissolved curcuminoids. C) Elution profiles of curcuminoids after first (ext.1), fifth (ext. 5), and tenth (ext. 10) extraction of 1 mg of solid curcuminoids. Also shown is the elution profile of the final pellet after ten extractions (residual) with 5% BSA. D) Same as in B, except that 50 mg of solid curcuminoids were sequentially extracted.

The differential solubility of curcuminoids was further examined by adding solid curcuminoids in amounts of 10, 20, 30, 40, and 50 mg to 5% BSA (Fig. 6A). The insoluble portion of the curcuminoids was pelleted and the supernatant removed and processed for concentration determination as described. The remaining insoluble curcuminoid pellet was reincubated with 1 ml of fresh 5% BSA and reextracted. This procedure was repeated a total of ten times. The amount of BSA-soluble curcuminoids declined most rapidly during the first three incubations. After the tenth incubation, the amount of curcuminoids soluble in 5% BSA declined to about 15–20% relative to the amount solubilized after the first incubation (Fig. 6A).

After the final tenth incubation, the remaining solid curcuminoid pellet was rinsed three times in water and dried under vacuum. The dried curcuminoids were dissolved in 200 μl DMSO and the concentration determined spectrophotometrically after serial dilution of aliquots in butanol. The total amount of curcuminoids in the remaining pellets had declined by only 3–17% following ten incubations with fresh 5% BSA (Fig. 6B). Much of this decline could be attributed to the removal of small amounts of suspended solid curcuminoids with the supernatant. When 10 μl of the DMSO-dissolved curcuminoids were added to 1 ml of 5% BSA, the amount of curcuminoids soluble in 5% BSA was again increased to concentrations ranging from 1500 to 2300 μM, which was consistent with the results observed in Fig. 3.

Sequential extraction of 1 and 50 mg of curcuminoids with 5% BSA was also monitored by reversed phase chromatography (Fig. 6C,D). These amounts were chosen to detect possible variations in individual curcuminoid levels due to different amounts of starting material available for extraction. After five extractions of 1 mg of curcuminoids, there was a sharp decrease in the relative amount of bisdemethoxycurcumin solubilized in 5% BSA. This decrease was even more pronounced after ten extractions. Concomitantly, there was an increase in the relative amount of demethoxycurcumin after five extractions, followed by a slight decrease after ten extractions. By comparison, the relative amount of solubilized curcumin increased dramatically after five extractions and remained largely constant thereafter, while the amount of total curcuminoids available for solubilization decreased. Moreover, while there was a moderate decrease in the amount of bisdemethoxycurcumin and demethoxycurcumin, the overall profile of the curcuminoids remaining in the insoluble pellet after ten extractions (Fig. 6C) was little changed from its original composition (compare Fig. 1C).

Following the repeated extraction of the larger amount of curcuminoids (50 mg), a different pattern emerged. While the relative level of bisdemethoxycurcumin declined over ten extractions, the relative amount of demethoxycurcumin increased after five extractions and remained constant thereafter. Compared to the extraction of 1 mg curcuminoids, the extraction of 50 mg resulted in a much lower increase in the relative amount of soluble curcumin. There was also less depletion of bisdemethoxycurcumin and demethoxycurcumin in the final pellet (Fig. 6D).

These results again confirm that the three curcuminoids exist in the preparation of solid curcuminoids in different solid forms, such that each curcuminoid is differentially available for solubilization in 5% BSA. The repeated extractions of either 1 mg or 50 mg of solid curcuminoids show that the bisdemethoxycurcumin was most readily available for solubilization, followed by demethoxycurcumin. When these respective sources were depleted, curcumin was available for limited solubilization, while the total amount of curcuminoids available for solubilization was declining. Nevertheless, even after 10 extractions the depletion of bisdemethoxycurcumin and demethoxycurcumin was not complete, since residual amounts remained in the final curcuminoid pellets.

It is conceivable that the curcuminoids exist as a mixture of pure and mixed crystals in the commercial preparations of solid curcuminoids. Since curcumin is the most prevalent curcuminoid in the mixture, this compound would be more likely to form pure crystals. It is tempting to speculate that mixed crystals are easier to solubilize than pure crystals, resulting in the preferential solubilization of bisdemethoxycurcumin and demethoxycurcumin from the crystalline state. The notion that crystal structure is a possible parameter in the solubility properties of solid curcumin is supported by the following observation. Solid curcumin (30 mg) was dissolved in 1 ml of acetone and allowed to completely evaporate at room temperature. The residual curcuminoids were then incubated with 5% BSA and the solubilized curcuminoids analyzed by reversed phase chromatography. The elution profile of curcuminoids solubilized from this solid (B: 23%, D: 33%, C: 44%) differed profoundly from that obtained from the original commercial preparation (B: 74%, D: 26%, C: 3%) [Fig 4B]. It is therefore conceivable that the evaporation procedure had altered the original crystal structure, thereby shifting the preferential solubilization in 5% BSA from bisdemethoxycurcumin to curcumin.

These results also suggest that BSA may form complexes with curcuminoids that differ in their binding affinities. For example, when DMSO-dissolved curcuminoids are first solubilized in 5% BSA, all high and most of the low affinity binding sites would be occupied. Consequently, upon further incubation with solid curcuminoids no additional solubilization would occur, since all available sites would already be occupied. In contrast, when 5% BSA is first incubated with solid curcuminoids, only the high affinity binding sites would be accessible for limited solubilization. The remaining lower affinity sites would then be available for subsequent solubilization of DMSO-dissolved curcuminoids, resulting in an additive response (Fig. 5). This concept can be extended to explain the different levels of solubility obtained by adding DMSO-dissolved or solid curcuminoids to FCS or BSA solutions (Figs. 2 and 3), and to account for the effects of sequential extractions of solid curcuminoids (Fig. 6).

### Curcuminoids are differentially soluble in sera from different mammalian species

**Figure 7:**
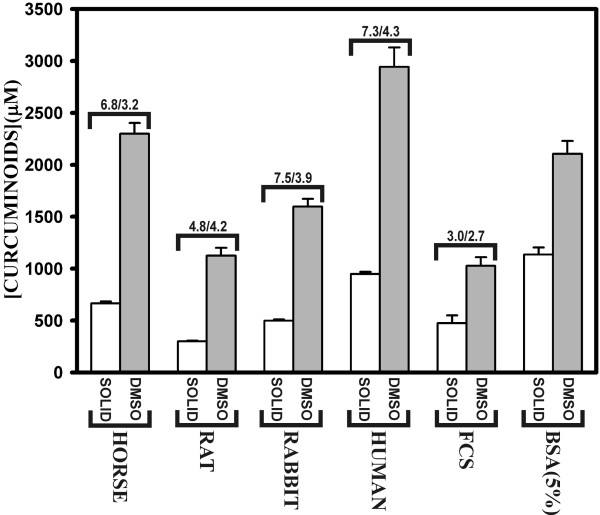
**Curcuminoid solubility in mammalian sera**. Either 30 mg solid (white bars), or 10 μl of DMSO-dissolved curcuminoids at a 500 mM concentration (gray bars) were added to 1 ml of horse, rat, rabbit, human, fetal calf (FCS) serum or 5% BSA solution. Brackets on top of the bars show total protein/albumin concentrations as provided by the supplier's data sheets.

Although albumin is a major mediator of curcuminoid solubility in serum, it is likely that other components also contribute. A comparison of sera from different mammalian species showed a high degree of variation both in total protein and albumin contents as well as their ability to solubilize curcuminoids. The highest level of curcuminoid solubility was observed in human serum followed by horse, rabbit, rat and fetal calf serum (Fig. 7). However, in general, curcuminoid solubility was poorly correlated with either total protein or albumin concentration. In addition, serum from whole rat and human blood prepared in this laboratory showed a 25–50% higher curcuminoid solubility than that obtained from the commercial source. Furthermore, curcuminoid solubility in human plasma was at least 50% lower than in serum (data not shown). This suggests that other factors, including the method of serum preparation or as yet unidentified individual or species-specific differences in serum composition, contribute to curcuminoid solubility. In addition, other serum components that were not investigated, such as lipoproteins, glycoproteins, and globulins, are also possible candidates for influencing curcuminoid solubility.

The effect of freeze-thawing sera on curcuminoid solubility was not systematically examined here. However, throughout the study, both FCS and BSA solutions were frozen and thawed multiple times without apparent effect on their ability to solubilize curcuminoids. Similarly, serum already containing soluble curcuminoids could be frozen and thawed without evidence of further curcuminoid precipitation or decline in biological activity.

### The solubilization of solid curcuminoids for cell culture applications depends on method of mixing

**Figure 8:**
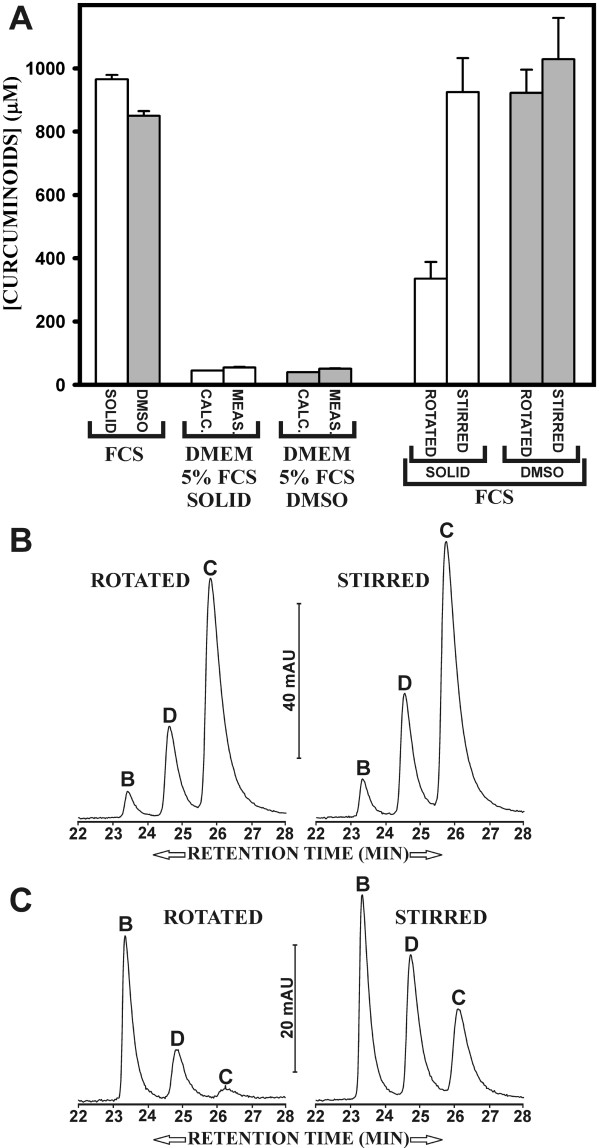
**Cell culture media preparation and curcuminoid solubilization in FCS produced by different mixing techniques**. A) Solutions for cell culture media were prepared by adding either solid (white bars) or DMSO-dissolved (500 mM) curcuminoids (gray bars) to FCS. The FCS was subsequently added to DMEM at a final concentration of 5%. The total curcuminoid concentrations in the media were then determined by butanol extraction. The measured (meas.) values were compared to the calculated (calc.) values (brackets) for both media prepared with solid (white bars) and DMSO-dissolved (gray bars) curcuminoids. On the right side of the panel DMSO-dissolved (gray bars) or solid curcuminoids (white bars) were mixed with FCS either by stirring or by rotation. The total amount of total curcuminoids solubilized by either method was determined spectrophotometrically. B) Elution profiles after separation by reversed phase chromatography of DMSO-dissolved curcuminoids solubilized in FCS, mixed either by stirring (right) or rotation (left). C) Elution profiles of solid curcuminoids solubilized in FCS, mixed either by stirring (right) or rotation (left).

Curcuminoids were solubilized in FCS for use in tissue culture medium to explore their effect on biological activity. Specifically, 29 ml of FCS were mixed either with 870 mg of solid or 145 μl of 500 mM DMSO-dissolved curcuminoids for 16–24 h with a magnetic stirrer. The final concentration of soluble curcuminoids was 966 μM for the preparation with solid curcuminoids and 850 μM for the preparation with DMSO-dissolved curcuminoids (Fig. 8A). Tissue culture medium was prepared by adding 25 ml of FCS-solubilized curcumin to 500 ml DMEM. Final curcuminoid concentrations were about 55 μM in the medium prepared with solid curcumin and 51 μM in the medium prepared with DMSO-dissolved curcumin. These measured values were within 10% of the calculated values expected from the dilution of FCS-solubilized curcumin (Fig. 8A).

Unexpectedly, the curcuminoid concentration achieved by stirring solid curcuminoids was about twice as high as that anticipated from the results described in Fig. 2. In those experiments, 1 ml of FCS was mixed with 30 mg of solid curcuminoids in 1.5 ml Eppendorf tubes by rotation on a Barnstead Thermoline labquake rotator for 16–24 h. This discrepancy in soluble curcuminoid concentrations produced by the two methods of mixing warranted systematic investigation. Therefore, 10 ml of FCS were mixed with 300 mg of solid or 50 μl of 500 mM DMSO-dissolved curcuminoids either by stirring or by rotation. Indeed, both mixing methods were similarly effective in solubilizing DMSO-dissolved curcuminoids in FCS (Fig. 8A). Conversely, mixing solid curcuminoids with 10 ml of FCS by rotation resulted in lower soluble curcuminoid concentrations than those produced by rotational mixing of 1 ml samples, even though the curcuminoid/volume ratios were the same. This could be due to less efficient mixing of larger volumes. In contrast, stirring solid curcumin produced an almost three-fold increase in the amount of FCS-solubilized curcuminoids (Fig. 8A). This procedure also resulted in physical changes in the suspended solid curcuminoid particles. Suspended curcumin obtained by rotational mixing could be effectively pelleted by low-speed centrifugation (3000 × g), whereas much of the stirred curcuminoids remained in suspension at this speed. This indicated that stirring resulted in a more effective dispersion of solid curcuminoid particles, thus making them available for more efficient solubilization in FCS.

Reversed phase chromatography of the DMSO-dissolved curcuminoids solubilized in FCS by the two mixing methods revealed no differences in the levels of individual curcuminoids (Fig 8B). The pattern was essentially the same as that produced by rotational mixing of volumes of 1 ml (compare Fig. 4B). Conversely, mixing solid curcuminoids by the two methods produced different curcuminoid concentration profiles in FCS. Rotational mixing effectively resulted in the same pattern as that described in Fig. 4B, whereas mixing by magnetic stirring provided higher soluble levels of demethoxycurcumin and curcumin. Nevertheless, in both cases bisdemethoxycurcumin remained the predominant component (Fig. 8C).

This demonstrates that particle size, resulting from different mixing methods is an important parameter in determining the relative solubility of individual and total curcuminoids. The effect of reducing particle size is particularly relevant when solubilizing solid curcumin. It is possible that other mixing methods not examined here, such as extended vortexing, could produce additional patterns of curcuminoid solubilization profiles. These results suggest additional parameters for differentially enriching specific curcuminoids in serum and cell culture media.

### The stability of curcuminoids in tissue culture media depends on storage and incubation conditions

**Figure 9:**
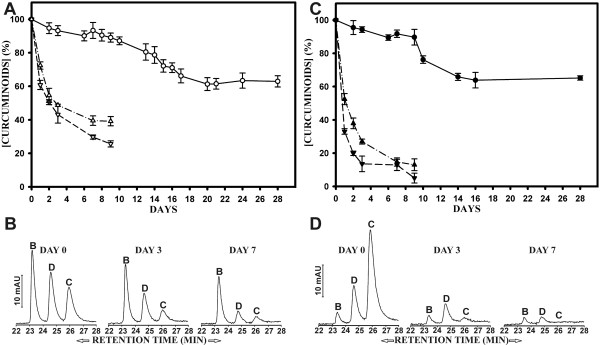
**Stability of curcuminoids in cell culture media**. A) Stability of curcuminoids in media prepared with solid curcuminoids. The media were either stored at 4°C (○) or in a cell culture incubator at 37°C, with (△) or without (▽) exposure to 5% CO_2_. B) Elution profiles after reversed phase chromatography of media prepared with solid curcuminoids at day 0, day 3, and day 7 of incubation at 37°C with 5% CO_2_. C) Same as in panel A, except that the media were prepared with DMSO-dissolved curcuminoids. The media were stored at 4°C (●) or in a cell culture incubator at 37°C with (▲) or without (▼) 5% CO_2_. D) Same as in panel B except that the media were prepared with DMSO-dissolved curcuminoids.

The stability of curcuminoids was investigated in cell culture media. Media prepared with solid and DMSO-dissolved curcuminoids were about equally stable when stored in a refrigerator at 4°C, both declining to about 70% of the original value during a four-week period (Fig. 9A,C). However, under tissue culture conditions at 37°C, the concentrations decreased rapidly within nine days to 30–40% for media prepared with solid curcuminoids (Fig. 9A) and to less than 20% for media prepared with DMSO-dissolved curcuminoids (Fig. 9C). In both cases the decline was more rapid when media were incubated without 5% CO_2 _(pH 8.0) [Fig. 9A,B]. It is likely that this was attributable to the lower pH of the media equilibrated with CO_2 _(pH 7.2). For cell culture studies, it is therefore advisable to change media daily.

The declining stability of curcumin in solution was defined as the loss of absorption at the wavelength of 427 nm. Curcumin was found to rapidly decompose in aqueous solutions, yielding the final degradation products vanillin, ferulic acid, and feruloyl methane [27]. These compounds have shifted their absorption maximum to the UV range and they no longer absorb at the wavelength of 427 nm. Thus, the decrease in absorption at 427 nm can be considered a reliable measure of the stability-related decline in curcumin concentration, in particular since it was accompanied by a proportional decline in biological effect on cells in culture (data not shown).

These conclusions were supported by reversed phase chromatography of curcuminoids from media incubated at 37°C in the presence of 5% CO_2_. In media containing serum prepared with stirred solid curcuminoids, the starting profile was the same as that shown in Fig. 8. The predominant component was bisdemethoxycurcumin with lower amounts of demethoxycurcumin and curcumin (Fig. 9B). During a seven-day incubation, all components declined in concentration. However, the most rapid decline was observed with curcumin and demethoxycurcumin. The most stable component was bisdemethoxycurcumin, which after seven days had declined by only about 50% (Fig. 9B).

In media containing serum prepared with DMSO-dissolved curcuminoids, the starting concentration of the individual curcuminoids (Fig. 9D) reflected the curcuminoid ratios present in the original powdered mixture (Fig. 1C). Curcumin, which at the onset was the predominant component, declined rapidly in relative concentration and became undetectable after seven days of incubation. The level of demethoxycurcumin declined less rapidly from a lower initial concentration. Bisdemethoxycurcumin was again the most stable component and its concentration declined moderately from what had been a very low initial concentration (Fig. 9D). In media prepared either with solid and DMSO-dissolved curcuminoids, the stability of the individual components declined in the order: bisdemethoxycurcumin > demethoxycurcumin > curcumin. The sum of the concentration declines of the individual curcuminoids observed by reversed phase chromatography (Fig. 9A,C) was in agreement with the values obtained by spectrophotometry of the total curcuminoids (Fig. 9B,D).

### The effects on cell proliferation of media prepared with solid and DMSO-dissolved curcuminoids are indistinguishable

**Figure 10:**
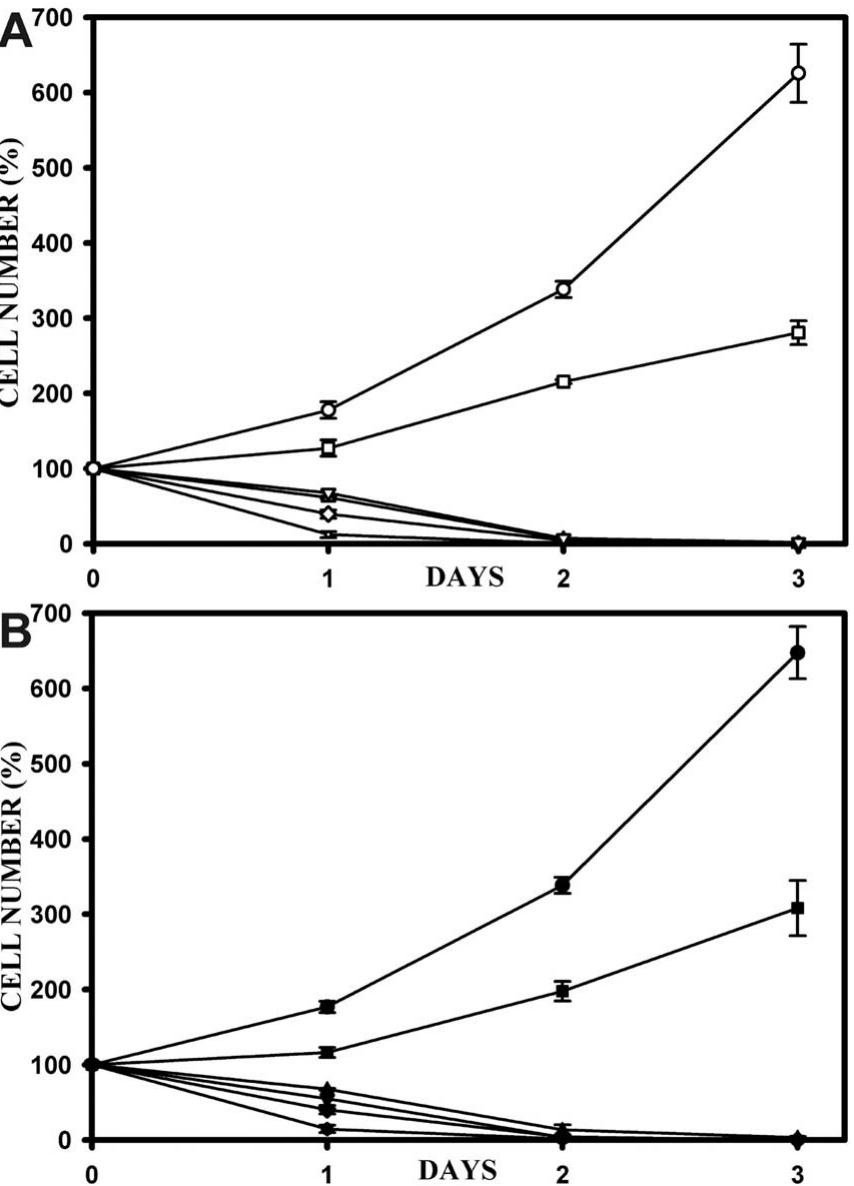
**Effect of curcuminoids on cell proliferation**. A) Cell survival (%) after incubating HeLa cells in media without curcumin (○) or with media prepared with solid curcumin at concentrations of 10 (□), 20 (▽), 30 (△), 40 (◇), or 50 μM (+). The initial seeding density of the HeLa cells was about 20% confluence for all cells, which was assigned the relative value of 100%. Cells were counted daily for three days and the data points represent average cell numbers from four different viewing fields with standard deviations (error bars). B) Same as in panel A, except that cells were incubated in media prepared either without curcumin (●) or with medium prepared with DMSO-dissolved curcumin at concentrations of 10 (■), 20 (▼), 30 (▲), 40 (◆), or 50 μM (●).

Media containing serum prepared with either solid or DMSO-dissolved curcuminoids were compared for their effect on cell growth and survival. The freshly prepared media were diluted with curcuminoid-free medium to 0, 10, 20, 30, 40, or 50 μM concentrations of curcuminoids. HeLa cells were seeded at a density of about 20% confluence (designated as a relative starting cell number of 100%) and then incubated at these curcuminoid concentrations for three days. The results were indistinguishable for media prepared either with solid curcuminoids or with DMSO-dissolved curcuminoids (Fig. 10). Without curcuminoids, HeLa cells divided exponentially with a doubling time of about 26 h. At a concentration of 10 μM curcuminoids cell division continued during the three days of incubation, although at a slower rate with a doubling time exceeding 48 h. At curcuminoid concentrations between 20 μM and 50 μM cell survival declined to zero after three days in a dose dependent manner (Fig. 10A,B).

In other reported cell culture studies, curcumin inhibited cell growth or induced apoptosis in a variety of cell lines at concentrations ranging from 10–100 μM [43-49]. The presented data concur with those results. Although the ratios of the individual curcuminoids were different in media prepared with solid or DMSO-dissolved curcuminoids, there was no detectable difference in their ability to inhibit HeLa cell proliferation under the culture conditions used here. Similar results were observed in a variety of cell lines [50]. These cell culture studies also suggest that curcuminoid levels required for efficient control of cell proliferation are vastly higher than those obtained in plasma by oral uptake [51].

## Conclusion

Methods are described that enable the solubilization of curcuminoids at high concentrations in serum or albumin solutions. The serum-solubilized curcuminoids can be quantitatively extracted with water-saturated butanol and their concentration determined spectrophotometrically at 427 nm. Once curcuminoids are dissolved, the serum can be diluted or concentrated at will without evidence of precipitation (data not shown). This allows for the preparation of cell culture media with precisely defined curcuminoid concentrations. The concentration of total curcuminoids that can be achieved in serum is about 100-fold higher than that required for inhibition of cell proliferation activity in cell culture systems [43-49]. This immediately suggests a novel method for curcuminoid administration by intravenous infusion of serum-solubilized curcuminoids, thereby circumventing the gastrointestinal tract. For example, if a typical human subject has a total blood volume of 5 liters, an intravenous infusion of only 50 ml serum would achieve an initial blood concentration of about 20–30 μM, an amount compatible with inhibition of cell proliferation in culture. Such defined high concentrations would also allow for the study of the metabolism and partition of curcuminoids into various fluid compartments to obtain optimal steady-state therapeutic levels of curcuminoids. The potential toxic effects associated with DMSO in these instances should be considered negligible, since the amount administered is a minute fraction of that considered acceptable in e.g. stem cell transplantation and blood progenitor cell cryopreservation [52,53]. However, the potential toxic effects of curcuminoids would have to be further investigated at such unprecedented plasma concentrations. Moreover, studies relating to the effect of curcumin on Alzheimer disease suggest that therapeutic or preventative effects could be achieved in as little as a 1–2 μM concentration range [23-25], which is less than 1/10 of that required for inhibiting tumor proliferation in cell culture. At these lower concentrations toxic effects are much less likely to occur.

In addition to providing generally high concentrations of soluble curcuminoids, the described procedures offer the opportunity to adjust the level of individual curcuminoids in serum preparations. For example, if solid curcumin is used to prepare serum, the predominant solubilized curcuminoid is bisdemethoxycurcumin, whereas curcumin predominates when DMSO-dissolved curcuminoids are used. Variations in mixing techniques for solubilizing solid curcuminoids in serum offer additional alternatives for maximizing solubility and modifying curcuminoid ratios. Furthermore, curcuminoids solubilized in sera by different methods can be combined to achieve optimal curcuminoid ratios for specific treatment regiments or cell culture applications.

## Abbreviations

FCS: fetal calf serum; BSA: bovine serum albumin; PBS: phosphate-buffered saline; DMSO: dimethylsulfoxide; APP: amyloid precursor protein; DMEM: Dulbecco's Modified Eagle Medium; OD: optical density; ATCC: American Type Culture Collection; PVP: polyvinylpyrrolidine; rt: retention time; mAU: milliabsorption units.

## References

[B1] Tayyem RF, Heath DD, Al-Delaimy WK, Rock CL (2006). Curcumin content of turmeric and curry powders. Nutr Cancer.

[B2] Jayaprakasha GK, Jagan Mohan Rao L, Sakariah KK (2002). Improved HPLC method for the determination of curcumin, demethoxycurcumin, and bisdemethoxycurcumin. J Agric Food Chem.

[B3] Tønnesen HH, Karlsen J (1983). High-performance liquid chromatography of curcumin and related compounds. J Chromatogr.

[B4] Duvoix A, Blasius R, Delhalle S, Schnekenburger M, Morceau F, Henry E, Dicato M, Diederich M (2005). Chemopreventive and therapeutic effects of curcumin. Cancer Lett.

[B5] Cui L, Miao J, Cui L (2007). Cytotoxic effect of curcumin on malaria parasite Plasmodium falciparum: inhibition of histone acetylation and generation of reactive oxygen species. Antimicrob Agents Chemother.

[B6] Shishodia S, Chaturvedi MM, Aggarwal BB (2007). Role of curcumin in cancer therapy. Curr Probl Cancer.

[B7] Karunagaran D, Rashmi R, Kumar TR (2005). Induction of apoptosis by curcumin and its implications for cancer therapy. Curr Cancer Drug Targets.

[B8] Singh RP, Agarwal R (2006). Mechanisms of action of novel agents for prostate cancer chemoprevention. Endocr Relat Cancer.

[B9] Sharma RA, McLelland HR, Hill KA, Ireson CR, Euden SA, Manson MM, Pirmohamed M, Marnett LJ, Gescher AJ, Steward WP (2001). Pharmacodynamic and pharmacokinetic study of oral Curcuma extract in patients with colorectal cancer. Clin Cancer Res.

[B10] Chattopadhyay I, Biswas K, Bandyopadhyay U, Banerjee RK (2004). Turmeric and Curcumin: Biological actions and medicinal applications. Curr Sci.

[B11] Maheshwari RK, Singh AK, Gaddipati J, Srimal RC (2006). Multiple biological activities of curcumin: a short review. Life Sci.

[B12] Thangapazham RL, Sharma A, Maheshwari RK (2006). Multiple molecular targets in cancer chemoprevention by curcumin. AAPS J.

[B13] Sharma RA, Gescher AJ, Steward WP (2005). Curcumin: the story so far. Eur J Cancer.

[B14] Aggarwal BB, Shishodia S, Takada Y, Banerjee S, Newman RA, Bueso-Ramos CE, Price JE (2005). Curcumin suppresses the paclitaxel-induced nuclear factor-κB pathway in breast cancer cells and inhibits lung metastasis of human breast cancer in nude mice. Clin Cancer Res.

[B15] Bachmeier B, Nerlich AG, Iancu CM, Cilli M, Schleicher E, Vené R, Dell'Eva R, Jochum M, Albini A, Pfeffer U (2007). The chemopreventive polyphenol Curcumin prevents hematogenous breast cancer metastases in immunodeficient mice. Cell Physiol Biochem.

[B16] Gao X, Deeb D, Jiang H, Liu YB, Dulchavsky SA, Gautam SC (2005). Curcumin differentially sensitizes malignant glioma cells to TRAIL/Apo2L-mediated apoptosis through activation of procaspases and release of cytochrome c from mitochondria. J Exp Ther Oncol.

[B17] Frank B, Gupta S (2005). A review of antioxidants and Alzheimer's disease. Ann Clin Psychiatry.

[B18] Ringman JM, Frautschy SA, Cole GM, Masterman DL, Cummings JL (2005). A potential role of the curry spice curcumin in Alzheimer's disease. Curr Alzheimer Res.

[B19] Frautschy SA, Hu W, Kim P, Miller SA, Chu T, Harris-White ME, Cole GM (2001). Phenolic anti-inflammatory antioxidant reversal of Aβ-induced cognitive deficits and neuropathology. Neurobiol Aging.

[B20] Garcia-Alloza M, Borrelli LA, Rozkalne A, Hyman BT, Bacskai BJ (2007). Curcumin labels amyloid pathology in vivo, disrupts existing plaques, and partially restores distorted neurites in an Alzheimer mouse model. J Neurochem.

[B21] Lim GP, Chu T, Yang F, Beech W, Frautschy SA, Cole GM (2001). The curry spice curcumin reduces oxidative damage and amyloid pathology in an Alzheimer transgenic mouse. J Neurosci.

[B22] Adlerz L, Beckman M, Holback S, Tehranian R, Cortés Tor V, Iverfeldt K (2003). Accumulation of the amyloid precursor-like protein APLP2 and reduction of APLP1 in retinoic acid-differentiated human neuroblastoma cells upon curcumin-induced neurite retraction. Brain Res Mol Brain Res.

[B23] Park SY, Kim DS (2002). Discovery of natural products from Curcuma longa that protect cells from β-amyloid insult: a drug discovery effort against Alzheimer's disease. J Nat Prod.

[B24] Ono K, Hasegawa K, Naiki H, Yamada M (2004). Curcumin has potent anti-amyloidogenic effects for Alzheimer's β-amyloid fibrils in vitro. J Neurosci Res.

[B25] Yang F, Lim GP, Begum AN, Ubeda OJ, Simmons MR, Ambegaokar SS, Chen PP, Kayed R, Glabe CG, Frautschy SA, Cole GM (2005). Curcumin inhibits formation of amyloid β oligomers and fibrils, binds plaques, and reduces amyloid in vivo. J Biol Chem.

[B26] Tønnesen HH, Másson M, Loftsson T (2002). Studies of curcumin and curcuminoids. XXVII. Cyclodextrin complexation: solubility, chemical and photochemical stability. Int J Pharm.

[B27] Wang YJ, Pan MH, Cheng AL, Lin LI, Ho YS, Hsieh CY, Lin JK (1997). Stability of curcumin in buffer solutions and characterization of its degradation products. J Pharm Biomed Anal.

[B28] Bernabé-Pineda M, Ramírez-Silva MT, Romero-Romo M, González-Vergara E, Rojas-Hernández A (2004). Determination of acidity constants of curcumin in aqueous solution and apparent rate constant of its decomposition. Spectrochim Acta A Mol Biomol Spectrosc.

[B29] Marczylo TH, Verschoyle RD, Cooke DN, Morazzoni P, Steward WP, Gescher AJ (2007). Comparison of systemic availability of curcumin with that of curcumin formulated with phosphatidylcholine. Cancer Chemother Pharmacol.

[B30] Garcea G, Jones DJ, Singh R, Dennison AR, Farmer PB, Sharma RA, Steward WP, Gescher AJ, Berry DP (2004). Detection of curcumin and its metabolites in hepatic tissue and portal blood of patients following oral administration. Br J Cancer.

[B31] Cheng AL, Hsu CH, Lin JK, Hsu MM, Ho YF, Shen TS, Ko JY, Lin JT, Lin BR, Ming-Shiang W, Yu HS, Jee SH, Chen GS, Chen TM, Chen CA, Lai MK, Pu YS, Pan MH, Wang YJ, Tsai CC, Hsieh CY (2001). Phase I clinical trial of curcumin, a chemopreventive agent, in patients with high-risk or pre-malignant lesions. Anticancer Res.

[B32] Sharma RA, Euden SA, Platton SL, Cooke DN, Shafayat A, Hewitt HR, Marczylo TH, Morgan B, Hemingway D, Plummer SM, Pirmohamed M, Gescher AJ, Steward WP (2004). Phase I clinical trial of oral curcumin: biomarkers of systemic activity and compliance. Clin Cancer Res.

[B33] Ravindranath V, Chandrasekhara N (1980). Absorption and tissue distribution of curcumin in rats. Toxicology.

[B34] Pan MH, Huang TM, Lin JK (1999). Biotransformation of curcumin through reduction and glucuronidation in mice. Drug Metab Dispos.

[B35] Péret-Almeida L, Cherubino APF, Alves RJ, Dufossé L, Glória MBA (2005). Separation and determination of the physico-chemical characteristics of curcumin, demethoxycurcumin and bisdemthoxycurcumin. Food Res Intern.

[B36] Hsu YC, Weng HC, Lin S, Chien YW (2007). Curcuminoids-cellular uptake by human primary colon cancer cells as quantitated by a sensitive HPLC assay and its relation with the inhibition of proliferation and apoptosis. J Agric Food Chem.

[B37] Hiserodt R, Hartman TG, Ho C-T, Rosen RT (1996). Characterization of powdered turmeric by liquid chromatography-mass spectrometry and gas chromatography-mass spectrometry. J Chromatogr A.

[B38] Jayaprakasha GK, Jagan Mohan Rao L, Sakariah KK (2002). Improved HPLC method for the determination of curcumin, demethoxycurcumin, and bisdemethoxycurcumin. J Agric Food Chem.

[B39] Huang MT, Ma W, Lu YP, Chang RL, Fisher C, Manchand PS, Newmark HL, Conney AH (1995). Effects of curcumin, demethoxycurcumin, bisdemethoxycurcumin and tetrahydrocurcumin on 12-O-tetradecanoylphorbol-13-acetate-induced tumor promotion. Carcinogenesis.

[B40] Barik A, Priyadarsini KI, Mohan H (2003). Photophysical studies on binding of curcumin to bovine serum albumins. Photochem Photobiol.

[B41] Verghese J (1993). Isolation of curcumin from Curcuma longa L. rhizome. Flavour Fragr J.

[B42] Paradkar A, Ambike AA, Jadhav BK, Mahadik KR (2004). Characterization of curcumin-PVP solid dispersion obtained by spray-drying. Int J Pharm.

[B43] Jee SH, Shen SC, Tseng CR, Chiu HC, Kuo ML (1998). Curcumin induces a p53-dependent apoptosis in human basal cell carcinoma cells. J Invest Dermatol.

[B44] Mukhopadhyay A, Bueso-Ramos C, Chatterjee D, Pantazis P, Aggarwal BB (2001). Curcumin downregulates cell survival mechanisms in human prostate cancer cell lines. Oncogene.

[B45] Dhandapani KM, Mahesh VB, Brann DW (2007). Curcumin suppresses growth and chemoresistance of human glioblastoma cells via AP-1 and NF-κB transcription factors. J Neurochem.

[B46] Deeb D, Jiang H, Gao X, Al-Holou S, Danyluk AL, Dulchavsky SA, Gautam SC (2007). Curcumin [1,7-bis(4-hydroxy-3-methoxyphenyl)-1-6-heptadine-3,5-dione; C_21 _H_20_O_6_] sensitizes human prostate cancer cells to tumor necrosis factor-related apoptosis-inducing ligand/Apo2L-induced apoptosis by suppressing nuclear factor-κB via inhibition of the prosurvival Akt signaling pathway. J Pharmacol Exp Ther.

[B47] Syng-Ai C, Kumari AL, Khar A (2004). Effect of curcumin on normal and tumor cells: role of glutathione and bcl-2. Mol Cancer Ther.

[B48] Balasubramanian S, Eckert RL (2007). Curcumin suppresses AP1 transcription factor-dependent differentiation and activates apoptosis in human epidermal keratinocytes. J Biol Chem.

[B49] Blasius R, Duvoix A, Morceau F, Schnekenburger M, Delhalle S, Henry E, Dicato M, Diederich M (2004). Curcumin stability and its effect on glutathione S-transferase P1-1 mRNA expression in K562 cells. Ann NY Acad Sci.

[B50] Sandur SK, Pandey MK, Sung B, Ahn KS, Murakami A, Sethi G, Limtrakul P, Badmaev V, Aggarwal BB (2007). Curcumin, demethoxycurcumin, bisdemethoxycurcumin, tetrahydrocurcumin and turmerones differentially regulate anti-inflammatory and anti-proliferative responses through a ROS-independent mechanism. Carcinogenesis.

[B51] Baum L, Cheung SK, Mok VC, Lam LC, Leung VP, Hui E, Ng CC, Chow M, Ho PC, Lam S, Woo J, Chiu HF, Goggins W, Zee B, Wong A, Mok H, Cheng WK, Fong C, Lee JS, Chan MH, Szeto SS, Lui VW, Tsoh J, Kwok TC, Chan IH, Lam CW (2007). Curcumin effects on blood lipid profile in a 6-month human study. Pharmacol Res.

[B52] Bakken AM (2006). Cryopreserving human peripheral blood progenitor cells. Curr Stem Cell Res Ther.

[B53] Windrum P, Morris TCM, Drake MB, Niederwieser D, Ruutu T, on behalf of the EBMT Chronic Leukaemia Working Party Complications Subcommittee (2005). Variation in dimethyl sulfoxide use in stem cell transplantation: a survey of EBMT centres. Bone Marrow Transplant.

